# Ethical and legal issues in medical practice

**DOI:** 10.4103/0970-1591.56191

**Published:** 2009

**Authors:** Joseph Thomas

**Affiliations:** Kasturba Medical College, Manipal, India

Rapid developments in the medical field in the last century have revolutionized the field of medical practice. It is now possible to diagnose diseases faster and more accurately using advanced diagnostic techniques. Medical management has become more effective with refined medications having more specific actions and fewer side effects. Surgical treatment has moved towards less invasive modes of management with lesser morbidity and faster recovery. Among all these developments, the medical profession in India is at crossroads facing many ethical and legal challenges in the practice of the profession. The medical fraternity is becoming more and more dependent on technology and market forces tend to influence decision making by the doctors. The fundamental values of medicine insist that the doctor's obligation is to keep the patients interest above everything else. The important issues of autonomy, confidentiality, justice, beneficence, and non malefecience are key factors that should guide the daily decision making by the doctor. These decisions may be involving a simple choice of antibiotics for an infection or the best medication for hypertension or hypercholestrolemia. It becomes more complex involving major ethical concerns in organ transplantation, clinical trials, genetic manipulations, end of life issues, or assisted reproductive techniques. However, the principles of ethics remain the same for all the above situations. The ethical guidelines of medical practice provided by The Indian Medical Council (Professional Conduct, Etiquette, and Ethics) Regulations, 2002 is aimed at strengthening the ethical standards among registered medical practitioners in India.

The health sector in India has seen a major transformation with health care becoming a profitable sector attracting investors from diverse and varied backgrounds with profitable motives. There is also an allegation that the practice of modern medicine is becoming more impersonal, and with the increasing dependence on technology, the cost of treatment also rises. It is a fact that cannot be ignored that there is increasing dissatisfaction on the part of the patients who are expecting more and more from the doctors, leading to increasing incidence of litigation. The Medical Council of India has a redressal mechanism that can give punishment to the erring doctor after proper investigative procedures. The unnecessary harassment of doctors who are falsely implicated in criminal negligence issues has been curtailed by the Supreme Court, which has issued guidelines for the criminal charging of a doctor for negligence.

The medical profession that was once considered noble is now considered along with other professions in the liability of paying for damages. The patients who wanted monetary compensation for the alleged medical negligence used to resort to the civil courts. This was the only avenue earlier that used to be a lengthy process with its detailed procedural formalities. The confusion about the inclusion of doctors under the Consumer Protection Act, 1986 has been laid to rest by the landmark decision of the Supreme Court in 1996 that puts the services of doctors for consideration under the purview of the Consumer Protection Act. This resulted in an increasing incidence of consumer cases where doctors were implicated for all types of allegations by patients. The recent Supreme Court guidelines that call for stricter evaluation by the Consumer Courts before proceeding with alleged medical negligence cases by the patients will be a boon to the doctors who will not be pulled into unnecessary litigation. However it has to be noted that the judicial bodies favour the patient who has suffered due to the negligent action of the doctors as reiterated by another Supreme Court decision recently confirming the decision of the State Commisison and giving a much higher compensation.

It is imperative that the present day medical doctors have continuing medico-legal education. Doctors have a legal duty to comply with the applicable ethical and legal regulations in their daily practice. Ignorance of law and its implications will be detrimental to the doctor even though he treats the patient in good faith for the alleviation of the patient's suffering. All actions that are done in good faith may not stand legal testing. With the increasing number of cases filed by aggrieved patients seeking legal remedy from doctors and medical establishments, it is no longer a matter of choice, but a context-driven legal mandate and necessity for the doctors to be conversant with basic legal issues involved in medical practice. This symposium aims at giving a basic insight into two main areas of medical practice:

1. The ethical issues in medical practice including changing doctor-patient relationships, the need for introducing ethical training in the undergraduate and postgraduate medical training, the modern challenges in urological practice, and the ethical and legal issues in kidney transplantation covered from an Indian perspective.
2. The legal issues covered include the basics of medical negligence, changing concepts of informed consent, and the practical issues of medical negligence cases with representative case decisions from the Indian Courts.

